# The Experience of China-Educated Nurses Working in Australia: A Symbolic Interactionist Perspective

**DOI:** 10.1371/journal.pone.0108143

**Published:** 2014-09-17

**Authors:** Yunxian Zhou

**Affiliations:** School of Nursing, Zhejiang Chinese Medical University, Hangzhou, China; UNSW Australia, Australia

## Abstract

**Background and Purpose:**

Transnational nurse migration is a growing phenomenon. This study explored the experiences of China-educated nurses working in Australia.

**Design:**

Using a constructivist grounded theory method, 46 in-depth interviews were conducted with 28 China-educated nurses in two major cities in Australia.

**Results:**

The core category emerged was “reconciling different realities”. Three phases of reconciling were conceptualised: realising, struggling, and reflecting. Realising refers to an awareness of the discrepancies between different realities. Struggling reflects the dilemma of the “middle position” and how being situated as “the other” is experienced. Reflecting is the process of making sense of the experience and rationalising the gains and losses associated with immigration.

**Conclusions:**

This study produced a theoretical understanding of the experience of China-educated nurses working in Australia. The findings not only inform Chinese nurses who wish to migrate but contribute to the implementation of more effective support services for immigrant nurses.

## Introduction

Transnational nurse migration is a growing phenomenon. One feature of this phenomenon is the global nurse shortage that is particularly dire in developed countries. In Australia, it is estimated that there will be a shortage of 109,000 nurses in 2025 [1]. International recruitment is seen as one strategy for addressing this problem. Because of its large labour pool, China is predicted to be an increasingly important source of nurse export [2], [3]. Statistics indicate that from 2004–05 to 2008–09, there were 14,950 registered nurses visa-sponsored to Australia, in addition to registered mental health nurses and midwives [4]. This is an increase from 2001–06 when 6,680 registered nurses migrated to Australia [5]. Although overseas-born nurses constitute one third of the nursing workforce in Australia and that percentage is expected to increase [6], relatively little is known about the experiences of immigrant nurses [7]–[9], particularly those from China. Thus, the purpose of this research was to explore the ways in which China-educated nurses construct meaning regarding the experience of working in Australia. The intent was to produce an in-depth theoretical understanding rather than a description of the experience. Two of the key concepts from this research have been published elsewhere [10], [11].

## Literature Review

The preliminary review of literature on the experience of overseas nurses was undertaken in 2007 and updated in 2014. Most studies located were from the UK and the major methodology adopted was qualitative [12]–[15]. A shared result from these studies is that most overseas nurses have a largely negative experience of working in another country. Some of the contributing factors include language barriers [13], [15]–[22], cultural issues [13], [16], [18]–[24], deskilling [13], [20], [25]–[26] and working relationship difficulties [13], [18]–[19]. These problems are exacerbated by a lack of support [13], [16], [18], [20], [27], a sense of isolation and alienation [13], [18], [20], [24], [28], experiencing racism and exploitation [8], [13]–[15], [20], [23], [27], [29]–[30], adapting to new expectations of the RN role [13], [15], [19], [21], [24], [31]–[33] and unequal opportunities [12], [14]–[15].

## Methods

### Design

The symbolic interactionist approach and, more specifically, the Chicago [34]–[35] and the Dramaturgical [36]–[37] schools underpin the theoretical perspective of this study. This perspective places particular emphases on meaning, interpretation, self, and social interaction. The details of this approach have been reported previously [11].

Constructivist grounded theory was the chosen method for this study [38]. Charmaz [38] emphasises meaning, context, and the interpretation of data. She also acknowledges that data collection and analysis are influenced by the researcher’s theoretical beliefs and interactions with participants. The constructivist view fits well with the premise of symbolic interactionism wherein research outcomes result from interpretation rather than the discovery of something that presumed to be *given* and *out there* [38].

### Ethic Statement

Ethical approval was granted by the Queensland University of Technology Human Research Ethics Committee in 2007. Prior to the interview, the study was explained to the participant and an opportunity to ask questions was provided. Written informed consent was obtained from each participant. Anonymity of participants was guaranteed by the development of a master list that identified participants by an assigned code. This list was kept separately in a locked filing cabinet away from the transcripts and audio-records. The researcher’s computer was protected by passwords. Only the researcher had access to the key and the list.

### Data collection and analysis

This research was carried out in Brisbane and Adelaide, Australia from 2007–2009. Purposive sampling was used from the outset to select participants who had received their basic nursing education in Mainland China and who had been working as registered nurses in Australia for at least 6 months. The main form of data generation was face-to-face in-depth interviews. The initial broad interview question was similar to the following: “*Tell me of your experiences of working as a registered nurse in the Australian health care system”*. Follow-up questions and probing questions were used to encourage elaboration of responses and to ensure clarity when necessary. After 28 interviews, study categories were tentatively established. Theoretical sampling was employed at this point with the intent of filling certain conceptual gaps. For this purpose, 18 of the initial 28 participants were invited for a second interview. This theoretical sampling process continued until there were no new emergent categories or sub-categories [39].

The interview language was Chinese, and data were generated by the author who is a Chinese nurse and was working for her Ph.D. in Australia at the time. The interviews were audio-recorded and lasted 40 to 158 minutes. After completion of each interview, the researcher wrote field notes to record non-verbal information about each encounter. Since the researcher was the data generation and analysis instrument, there was some imperative to consider how researcher subjectivity could influence the research findings [40]. For that purpose, a reflexive journal was kept to record the impressions, thoughts, problems, and decisions generated during the research process and the rationales underlying these decisions. The journal was included as a data source and as a contribution to theoretical sensitivity during analysis. It was also used as a strategy to achieve transparency and to ensure rigour in the research process. Finally, the literature was consulted as another source of data to expand understanding.

Based on grounded theory methods [38]–[39], we began data analysis immediately after the first interview using the constant comparison method. This data analysis strategy entails the following series of reiterative coding steps: initial coding, focused coding, and theoretical coding [38], [41]. A core category that accounted for the maximum amount of variation in the study phenomena was identified, and all other categories were integrated around this core category. Coding was conducted in Chinese. In addition to coding, the researcher wrote memos during the analysis to record her thoughts about the coding and questions and directions for further data generation. Theoretical rigour and procedural rigour were employed to ensure the rigour of this study. The details have been reported elsewhere [11].

## Findings and Discussions

All participants were female ranged in age from 20 to 50 years old. The participants’ nursing experience before immigrating to Australia ranged from 1 to 20 years. Among the participants, 24 had worked as staff nurses, 2 as nurse managers, and 2 as lecturers in nursing schools in China. With the exception of one participant, who was working in a nursing home, all participants were employed in hospital settings. The durations the participants had been working as registered nurses in Australia ranged from 6 months to 4 years.

**Figure 1:**
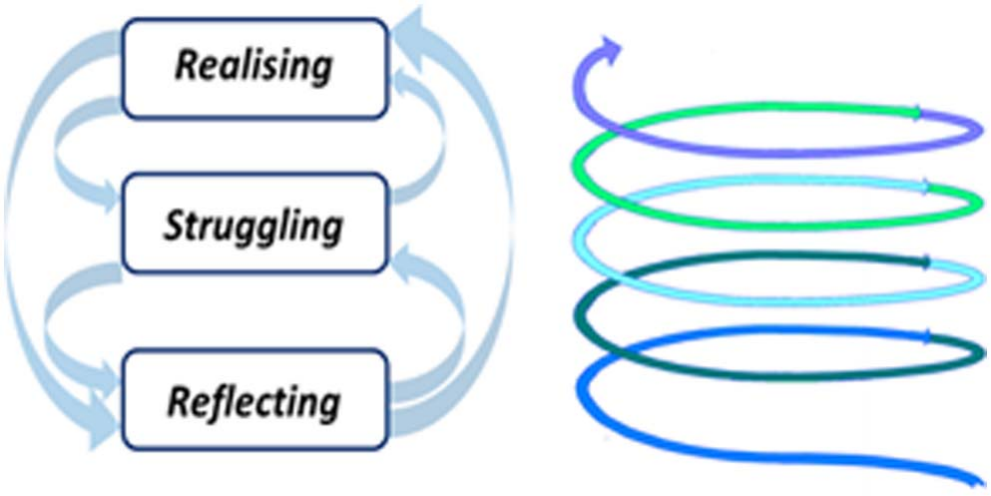
The core category of reconciling different realities.

The overall experiences of the participants are outlined in Table 1. The core category of this study was “reconciling different realities” (Figure 1). The term “reconciling” was not literally used by the participants to communicate how they experienced immigration. Rather, the term was derived from multiple descriptions, comments, and ideas expressed by the participants about the changing realities and the process of making congruent, letting go, and reframing unpleasant experiences after immigration. The “different realities” were socially constructed. While growing up in China, the participants had acquired a meaning system for interaction through socialisation. These meaning systems were shared by other Chinese people and reflected the social fabric of China. On arrival in Australia, the participants encountered different systems of meaning and thus different views of reality. Therefore, reconciling was a large part of the immigration experience for these nurses. Three phases of reconciling were conceptualised: realising, struggling, and reflecting. What follows is a depiction of each phase in the form of a category and its sub-categories.

**Table 1:** The overall experience of China-educated nurses working in Australia.

Core category	Categories	Sub-categories
Reconciling different realities	Realising	It is indeed different
		This is the Western way
		You are you and I am I
	Struggling	Caught between two worlds
		You have a lot to learn
		This is your own business
	Reflecting	A sense of loss
		Reconstructing the self
		It is hard to go back

### Realising

#### It is indeed different

In Australia, nursing is considered to be an independent profession, and nurses are not overtly subservient to doctors. The organisation of nursing work encourages some degree of autonomous care, and nurse managers are generally not pre-occupied with directly governing the nurses. As a result, the participants noted that nurses in Australia were required to make more independent decisions than nurses in China.

*Generally speaking, nurses here are more independent. They don’t rely on doctors totally. They can have their own thoughts and make decisions on the caring of patients. (Participant 4, Interview 4)*

While decision-making implies greater power and autonomy, the participants were initially uncomfortable with the increased accountability for their decisions. They feared that their nursing knowledge might be inadequate and that they needed more knowledge to better cope with the increased level of autonomy. It is probable that this was a perceived rather than actual knowledge deficit. The nurses may also have reverted to novice roles in the new context and thus looked for decision-making rule. A further reason our participants were concerned that their knowledge may have been inadequate was related to their foreign status. They were reluctant to make decisions because they expected that no protection would be provided if they made an incorrect judgment.

One example of the differences in nursing practice between China and Australia is related to basic nursing care. For the participants, basic nursing care constituted a large component of the nursing work in Australia.

*As to nursing in Australia, I found nurses here were doing too much. They do much more basic nursing care than we used to in China. (Participant 5, Interview 5)*

Nurses usually perform technical nursing such as injection, cannulation, and medication administration in China. The reality that nurses do not typically provide direct care in China has several dimensions. First, nursing departments are often understaffed, and nurses are unable to meet the basic care needs of patients. Second, there is the moral obligation to look after sick family members in Chinese culture [42]. Meeting basic needs by families or personal carers through the provision of direct care is seen as a way of demonstrating care and affection [43]. Third, nursing is considered semi-professional, and nurses are of low social status in China. The media portrays nurses as people who do little other than the “hard and dirty work” [44]. Distancing oneself from basic nursing care is considered a strategy for protecting the professional integrity of, and gaining social respect for, nurses in China.

Initially, it was difficult for the participants to accept the washing, toileting, and feeding patients as parts of a nurse’s role in Australia.

*I swore that I would never work there again after 6 weeks of clinic practice in the medical unit. That was too much. I have never done anything that dirty and that tiring in my nursing life. (Participant 22, Interview 22)*

The participants experienced tension between being a professional and performing tasks considered to be “unprofessional” such as washing, toileting, and feeding patients. This type of nursing work would usually be carried out by untrained workers or family in China and therefore viewed as unskilled and carries stigma. The participants were hesitant to inform families and friends in China that they were performing such tasks in Australia for fear of embarrassment.

*I feel too embarrassed to tell people (the fact)…If I tell my family that a nurse in Australia needs to shower the patient, I think even my family would find it very hard to accept. (Participant 16, Interview 16)*

To cope with the tasks of basic nursing care, the nurses separated themselves from what the work implied of them (i.e., low status). The participants also rationalised the performance of these tasks to others and themselves. Part of this strategy entailed reframing the meaning of basic nursing care by focusing on the fact that the tasks the participants were performing were beneficial to the patients and their family members. The reality that the local nurses willingly performed the tasks of basic nursing care helped the participants to reconstruct their perceptions of basic nursing care. The appreciation of, and positive feedback from, patients further reinforced the legitimacy of this new meaning. Using the reactions of others, the participants reshaped their views of basic nursing care.

*It is not a wrong thing for nurses to provide basic nursing care as nursing should be human centred. (Participant 7, Interview 42)*

#### This is the Western way

In addition to the changes in the nature of nursing work, the ways nursing care was delivered also changed. It appeared that the local nurses concentrated more on communication, while the participants were more concerned about “real nursing work”.

*I don’t know what they (local colleagues) are doing, chatting with doctors–a waste of time from my perspective. (Participant 26, Interview 40)*

The concept of real nursing work is constructed in a particular social context. In China, the implementation of the market economy delineates nursing care as a commodity with a price attached. Physical labour is the most visible aspect of nursing care in the sense of paid work because one is seen to be doing something. In contrast, the invisible nature of *soft* nursing work (i.e., emotional labour of caring) renders its cost imperceptible and economic compensation difficult. This definition of real nursing work shaped the communications between the participants and patients, which were usually brief, predominantly task-oriented, and concerned with physical care.

The participants not only communicated less but also communicated differently. For example, due to a lack of understanding of English expressions, requests were often articulated through direct Chinese translations that often sounded, and were perceived as, rude and impolite by colleagues.

*Although she (a Chinese nurse) did a lot of physical work, she talked to colleagues like this: I do this and you do that. Colleagues perceived this as an order…and not being given a chance to express their opinions…They felt annoyed being told by a newcomer. (Participant 14, Interview 14)*

Language is the symbol used for communication which stands for shared meaning within a given community [35]. From the symbolic interactionism perspective, the expression of politeness is socially defined and not a self-existing entity with an intrinsic nature [34]. Once the consensus of politeness has been agreed upon, it is taken for granted as a routine by members of a community to sustain social order. People who conform are considered polite while those who breach the rules are seen as acting impolitely. The participants acted on the basis of their previous understandings of politeness, were neither relevant nor appropriate when applied to their new contexts. Problematic situations required the nurses to reflect on the definition of the situation and construct new actions instead of responding in pre-established ways. One such example is that the nurses found it necessary to appear warmer, softer and more suggestive in posing requests.

*When communicating with others, I tried to learn how to be polite so as to be more acceptable by others. Sometimes, my words sound too formal and impolite, but I found others can put it in a softer and more acceptable way. (Participant 12, Interview 31)*

Differences in communication were also reflected in how nurses addressed patients in Australia.

*Here every nurse calls everyone sweetie, love, things like that. It is totally different from us… I have never thought of addressing a patient so intimately. It is hard for us because we don’t feel this way. (Participant 20, Interview 41)*

Such norms of addressing others are also socially constructed. In Chinese culture, words such as *sweetheart* and *love* are used only when people address someone close and usually in private [45]. To be explicit with someone who is not close sounds not only unnecessary but also disingenuous [45]. The “Australian” form of addressing a patient was alien to the participants. However, the nurses saw the need to do so to conform to the social norm of the work setting.

*…but I think gradually we need to learn from local nurses. Anyway, do in Rome as the Romans do…As an Asian, local people will regard you with special respect when they hear you speak good English and see you behave like an Australian. (Participant 20, Interview 41)*

The participants learned to be “on” and perform for others. These performances functioned to create and sustain a “projected self” that appeared normal and allowed the participants to be viewed as legitimate [36].

#### You are you and I am I

By immigrating to Australia, the participants relinquished previous social ties in China. While seeking to build new relationships in Australia, the nurses encountered many barriers. First, participation in social activities required the participants embrace local cultural norms. However, living in Australia brought forth the incongruities between personal and social values. The time away from home, combined with exposure to the experiences of colleagues, allowed the participants to re-examine their own beliefs and practices. This seemingly insurmountable difference gave rise to the feeling that, “*we cannot live lives like that”* in the participants.

In addition, without common experiences, meaning is not readily shared, which makes joint action problematic and community building difficult. Any conversational topic was usually unfamiliar and uninteresting to our participants.

*I know I should try my best to communicate more with others. But sometimes I am too tired to talk with them. I always feel I don’t have much to talk about. (Participant 2, Interview 2)*

There was always a risk of embarrassment due to inadequate engagement in social activities. Although cherishing the chance to get to know people, social activities can lose the intended meaning for the participants.

*I also attend some social activities among colleagues. But in this atmosphere, I am not truly there to relax and enjoy that atmosphere. I go for the sake of going. (Participant 17, Interview 34)*

The social psychological distance between the participants and their colleagues functioned as an invisible wall that resulted in feelings that “*we are among but we are not in”* in the participants. As a result, the participants perceived collegial relationships in Australia as superficial and existing only at the level of a working relationship.

*Sometimes they (local colleagues) are very polite and they are used to act like that. However…I think they are more being polite than affective…They don’t really care about you. (Participant 12, Interview 12)*

It appeared that the participants’ colleagues communicated out of courtesy rather than genuine interest and that this courtesy sustained the disconnection experienced by the nurses. The ideology of individualism, which prevails in Australian society, implies a preoccupation with the self and loose human connections. The comparison of human relationships in Australia to those in China exacerbated the perception that colleagues were courteous but not close.

*Anyway, they (local colleagues) come when they are on duty and they leave when they finish the shift. In China, we are colleagues even after the work. We go out together, and then we become very good friends. (Participant 1, Interview 1)*

Indeed, relationships with colleagues did not go beyond ritual greetings. The greetings themselves were not only evidence about the relationship but were the entire relationship.

### Struggling

#### Caught between two worlds

There was tension between the participants’ desire to hold on to their old selves and the need to conform to the new society. The Chinese element such as conformity, connectedness, and interpersonal values of the collectivistic systems was deeply embedded in the minds of the participants while growing up in China. Upon relocating to Australia, the nurses saw a clear need to fit in.

*Because you live in the real society here, not in a dream, your life has to change in some way after immigration. How you work, how you entertain, how you make friends, all of these have to be changed in order to carry on with your life. (Participant 1, Interview 43)*

Two strategies for fitting in were conceptualised in this study: “negotiating boundaries” and “switching off”. Negotiating boundaries indicates the making of conscious decisions regarding how far the participants will fit in.

*I pick up something and leave out others…It is like I put those things I consider suitable for me into my ‘personal basket’ on the way while leave out others. (Participant 6, Interview 28)*

Because it is hard to ‘assimilate’ completely [46], the China-educated nurses adopted rules for fitting in at the behavioural rather than the value level [10]. It is possible that some element of integration also occurred. Here, the use of the term ‘assimilate’ reflects the wording used by the participants about the pressure to fit in, the reality that any changes would have to be made by the participants, and to contrast experiences with the political rhetoric of integration. The external behaviour of the participants changed such that they did not appear too distinct in the new society. In broad terms, negotiating boundaries also means that the participants adopted some values and practices but not others and did so to differing degrees [10]. By negotiating boundaries, a delicate balance was constantly constructed.

Switching off indicates behaving differently within different contexts. In the public sphere, Western culture shaped the behaviour of the participants, while in the private sphere, Chinese tradition dominated [10].

*I don’t have local friends and I don’t want to either. The involvement with locals at work is unavoidable. As to my life, I don’t want it to be so. (Participant 22, Interview 22)*

Bun [47] stated that the strategy of switching off is reflected by the metaphor of one face, many masks; for our participants, this meant being Chinese now and not being Chinese later depending on the situation. One reason for alternating between ‘masks’ is that the workplace offered the participants little room to be Chinese. However, in their home lives, the participants still had a choice because their home lives were personal business. In a broader sense, switching off was also adopted when the participants travelled to China from Australia. The participants experienced a need to conform to Chinese culture and act accordingly while visiting home. Upon their return to Australia, it was necessary to revert back to being less Chinese.

The intermediate positions of the participants also gave rise to an identity dilemma regarding whether to be Chinese or Australian. Indeed, the transformation of cultural identity is not an easy undertaking [48].

*Sometimes I was caught in a dilemma. Because on one hand, I want to fit into the society here; on the other hand, I don’t want to completely lose my true self. (Participant 3, Interview 3)*

From SI perspective, cultural identity is socially constructed. It is often asserted through a process of exclusion where feelings of belonging depend on being able to say who does not belong [48]. In many respects, the Australian identity prevented the participants from identifying with Australia. The Australian individuals did not actively exclude the participants; rather, the existence of the Australian identity created boundaries that marginalised the nurses. This explains why the participants felt not so much that they were actively marginalised but that they simply could not be Australian.

*After all we have grown up in China and we received our education from China and we have been influenced by Chinese culture. We don’t feel we can be Australian. (Participant 17, Interview 34)*

Identity claims also depend on others [49]. In other words, people know who “they” are in relation to the other [35]. The significance of this concept is reflected in Cooley’s [50] metaphor of the looking-glass self where we often see our reflections in the eyes of others and even imagine what they think of us. If Australians think of China-educated nurses as “foreigners” (because of physical appearances and/or accents) and people who, despite living in Australia do not belong to Australia, then the nurses will internalise and reflect upon the way others view them (that is foreigners as outsiders).

*I am still considering whether I should change my passport or not. Even if I do make the change, local people will not think of me as an Australian because of my physical appearance. (Participant 15, Interview 30)*

This uncertainty about acceptance created ambivalence for the China-educated nurses regarding their place in Australian society [10]. The resultant feelings of alienation caused the participants form communities with other Chinese people and live a Chinese life overseas.

#### You have a lot to learn

When the participants first began working in Australia, everything appeared new, and there were many unknowns. One such unknown was the informal use of English.

*Sometimes colleagues are telling a joke and things are very humorous. Yet we are wondering what the meaning of the joke is? Why everyone is laughing? (Participant 18, Interview 18)*

Just as identity is socially constructed, so is language [35]. The meanings inherent to informal language are established through long-term usage and are thus highly nuanced and contextual. A common ground is necessary to appreciate a joke, and this common ground was not present for the participants. Moreover, verbal communication becomes more of an issue during clinical emergencies. The mind works slower and requires more effort in these situations when operating in a second language. Additionally, colleagues usually become less patient and talk more quickly in emergency situations.

As Xu [51] indicated, language barriers have a range of implications. The participants tended to remain silent during interactions for fear of being ridiculed. Furthermore, the inability to speak out served to reinforce the stereotype that Chinese nurses are shy, unassertive, and not equipped to be leaders.

*When your language is not good, you appear stupid and people look down upon you. (Participant 23, Interview 23)*

Tacit knowledge was another aspect of the unknowns faced by the participants.

*When people here getting old, they may choose to go to a nursing home. We have no idea of the steps involved in this process…This is considered common sense to locals…They know it. But to me, I never know it before I go through it. (Participant 10, Interview 10)*

Although hospitals have clear policies about many procedures, information that is taken for granted by locals is often not made clear to non-locals. In addition, something that is common sense in one culture may not be common sense in another. An Australian local might have no idea about what is problematic for an immigrant. Thus, it was often difficult, although not impossible, for the participants to develop thorough understandings of tacit knowledge.

Additionally, differences in skin colour, language and culture resulted in the participants being labelled as “the other”.

*They think your skin colour is yellow, you are Asian and you are Chinese, so they feel uncomfortable with you and they treat you as a foreigner and will arrange critical patients to you in your work. (Participant 26, Interview 26)*

Otherness provides one a reason for dislike and the lack of desire to understand. Being the other also means not being accepted and recognised by peers as a valued and contributing member. Due to this “looking-glass” [50] of their colleagues, the participants were aware of their own feelings about the views of their colleagues. A sense of shame may result because the participants were aware they were less desirably evaluated by other. This emotion, in turn, became a powerful motive for the participants to learn to improve themselves. Thus, it seems that learning was a central feature of their lives.

#### This is your own business

While the desire to appear competent and not lose respect motivated learning, it also rendered it difficult to disclose unknowns and seek support. The participants concealed or underplayed their doubts and sought to work as normally as possible.

*I would try to be “smart” on something. I wouldn’t say I didn’t know. However I would observe local nurses nearby and see how they did it. Sometime I feel it is necessary to act this way as people would otherwise suspect your qualifications if you said you didn’t know something which might consider common sense to them. (Participant 12, Interview 31)*

This concealment was a rejection of the social significance of the problem and not a denial of the problem per se. There are certain risks associated with not asking questions, such as patient safety or having the lack of understanding revealed anyway. As a result, the participants prioritised what they extend control over, what they disclosed, and whom they disclosed it to based on immediate clinical significance.

*I need to have my own judgment. If the question is something that I can learn at home by myself then I just keep it to myself. If it is urgent, serious, or there is potential risk involved, I have to ask colleagues immediately. (Participant 6, Interview 28)*

The anticipation of colleagues’ responses shaped the participants’ actions. It seems that this strategic disclosure was for self-protection purposes. In Goffman’s [36] terms, it was a form of impression management. Through self-presentation, the participants sought to convey the impression that they were competent nurses to others and to be consistent with the overall social expectation.

The participants also experienced a loss of social support after immigration. This was further exacerbated by the initial lack of social networks in Australia. Additionally, regarding emotional support, both families and colleagues were considered unhelpful. The participants assumed that fellow Chinese people with similar experiences could better understand their experiences. They also perceived it to be less shameful to ask for support from other Chinese people. However, support from fellow Chinese people was minimally utilised because the participants did not want to be perceived as a burden to others.

*Each Chinese in Australia has his or her own life stress…Since all of us are not easy, we try our best not to bring extra trouble to others. (Participant 3, Interview 3)*

Additionally, the support provided by the workplace was inadequate and inconsistent. Moreover, the participants expressed concern about the meaning of support.

*I don’t think it is necessary for the hospital to provide extra support for us… If we needed an extra training program, that would render us disadvantaged rather than benefit us. It may cause people to think that we are inadequate and that we lack something. (Participant 7, Interview 7)*

That few participants expected support might also indicate that they were socialised to take responsibility for themselves. Because they were employed as qualified nurses, the participants wanted to demonstrate their abilities and to be seen as responsible. In a society that values independence and self-reliance, the nurses considered it natural to be self-sufficient and endure hardship. Thus, the participants thought that what was unknown to them was their responsibility, which caused that the lack of support remained unquestioned.

### Reflecting

#### A sense of loss

Immigration removed the participants from many relationships and predictable contexts. Leisure activities differ in Australia, and such activities are not readily available and affordable. Without families and friends and in the absence of a social network in the new community, life was boring and homesickness was constant. The unfamiliar environment explains, in part, the inconvenience of Australian life. The participants had no idea what was appropriate in Australia. Rather, every minor detail required considerable effort [52]. This loss of “at-home-ness” translates into a sense of uprootedness, alienation, and insecurity [52].

Additionally, feelings of loss were not limited to the participants but extended to their immediate families. Most husbands experienced difficulties finding appropriate employment in Australia.

*Here, most Chinese men cannot find right job. This is a common issue faced by migrant nurses. For some men, even if they had important jobs in China, they won’t find anything at all to do here if their language is not good enough. (Participant 12, Interview 12)*

George [53] described the experience of men in this position from two perspectives, the loss of status compared to their wives and the loss of status with respect to their prior social position. Work is not just a means of living; it is a vital source of one’s self [54]. The husbands lost an important component of their identity as main providers for the household. Traditional Chinese family values maintain that the man’s place is “outside” the family and that the woman’s responsibilities lie “inside” that family, which makes role reversal difficult [10]. Conventional gender roles may partially reverse for men and women as they re-negotiate domestic labour and child care [53]. It is also possible that dependent men may feel their gender identity is threatened and are therefore less likely to do “women’s work” in the home [53]. The ongoing connections to China and Chinese community may also accentuate existing gender hierarchies. These changes in family dynamics have the potential to give rise to family conflicts over gender relations [10] and even cause marriage breakdown. These divergent interests within families bring ambivalent feeling to some participants.

Because of the poor employment prospect, finding the right partner to marry in Australia was a concern for single nurses, and many were uncertain about their future. The traditional Chinese view is that marriage is indispensable for securing a future and creating a sense of home [10]. Failing to get married reinforces a sense of rootlessness. Nonetheless, immigration changed the nurses’ perceptions of marriage. They considered it unwise to marry Chinese men who worked in China because of the poor job prospects for those men in Australia. However, due to differences in values and worldviews, marrying a western man living in Australia was also undesirable. The nurses had to confront this at some point in time. Moreover, in addition to issue regarding prospective partners, marriage choices also involved issues related to future living arrangements.

An additional form of loss was the loss of career opportunities. The participants perceived that they were much unlikely to rise to managerial positions despite their relatively superior qualifications and greater seniority.

*In China, your sense of value, and how to say, you may work in a respectable position, you may be promoted to a high position. It is hard to achieve this here. (Participant 17, Interview 17)*

The nurses perceived that the higher the nurse level, the higher the requirement in terms of language and cultural skills. Thus their career development opportunities were constrained because of inadequate language skills and lack of familiarity with the Australian system.

*Because of the constraint of English language, there are some limitations for us on promoting to higher level nurses such as clinical nurse, nurse practitioner or nurse educator. The limitation is there. (Participant 25, Interview 38)*

Interestingly, the idea that there was a lack of opportunity for promotion was constructed by the participants as their own problem, rather than an institutional issue [11]. As immigrants, many participants assumed their position was disadvantageous. They felt that their language skills were insufficient to build a career in Australia and that they could only have a job [10]. Some believed that promotion was still possible but dependent upon hard work and individual improvement.

*As to promotion and career development, I think there are many opportunities here as long as you are capable enough, willing to show your ability, being confident about yourself, fluent with English, and communicate well with doctors and nurses…I think it is an issue of your personal capability and language skills. (Participant 20, Interview 41)*

This attitude reflected their coping strategies and resilience in the response to the ambiguities surrounding discrimination. As Larsen [55] argued, for psychological reasons, the nurses may have denied the centrality of institutional discrimination and resisted its destructive effects. By explaining their experiences as their own inadequacies (which are apparent and readily acceptable anyway), the participants managed to motivate and improve themselves and to maintain hope and aspirations despite barriers [55]. This capacity for meaning-making provided the nurses with a sense of agency and some level of control over their situations.

#### Reconstructing the self

After immigration, the changes in the realities of the participant meant that they had left behind aspects of their previous selves and possibly the senses of pre-eminence they once enjoyed in China.

*In China I can easily be the excellent one…But here I feel I cannot be as good as them however hard I try. I feel I am only average…It is hardly achievable to be better than locals…Also the advantage once I possessed is no longer there. (Participant 12, Interview 12)*

The previous advantages of the participants disappeared, and their past accomplishments were erased as if they had never happened. The nurses began to realise that they were no longer who they had been and that they needed to learn from others. The result was that the participants became more humble and less ambitious.

*I felt each of us was born to be proud inside… But when you go abroad, you put yourself very low….You start by learning how to walk. You become humble after that. (Participant 10, Interview 37)*

Several interactionist concepts are relevant to explaining how individuals reconstruct the self. Mead and Morris [35] stated that “taking the attitude of the other” towards one’s own conduct is the essential characteristic of social conduct. Other concepts such as “reflected appraisal” and the “looking-glass self” [50] assume that our perception of the self grows from our interactions with others. As Cooley [50] argued, we form our views of ourselves on the basis of how others see us and how others react to us. The China-educated nurses were always “receivers of knowledge” and thus came to believe that they were less competent, which undermined their confidence.

The obvious foreign status also brought a sense of vulnerability. In some instances, the participants were uncertain whether they were the target of racism or discrimination. Most people in Australia were polite but some behaved in a hostile manner towards the nurses.

*Sometimes we were bullied by the agency because of our foreigner status…That is to say, they thought since they sponsored permanent residency for us, we had to accept any shift as a return…Sometimes I was threatened that my visa would be cancelled if I didn’t work the shift they demanded. (Participant 8, Interview 8)*

The participants shared the perception that as Chinese nurses their mistakes were more visible.

*As a foreigner, I work very carefully …You will encounter people who label you as Chinese, a “Chinese” nurse… I feel it is going to be a different story for a Chinese nurse to make the same mistake compare to a local nurse. (Participant 12, Interview 12)*

The participants worried that being Chinese may provoke a more punitive response if trouble did arise. When an Australian nurse made a mistake, responsibility for that mistake was attributed to the individual; when a China-educated nurse made a similar mistake, the mistake was reported in such a way that responsibility for the error was shared by the whole immigrant population.

Although immigration is stressful, it also enhances growth [56].

*The hardship of immigration made me grow up…My sense of values changed. I pay less attention to material wealth and being more genuine in interactions with others. (Participant 9, Interview 32)*

A sense of personal growth arises out of hardship and challenge, and this growth is often overlooked in the literature. Despite the difficulties and unmet support needs, the participants demonstrated great strength and resilience. The struggling was painful but also, on reflection, rewarding. The participants became more mature, stronger, and more independent. Throughout the immigration experience, the participants ascribed meaning to what they had experienced and focused on the broader picture, remaining hopeful while enduring the circumstances of the present.

*Life abroad is cruel and to survive is cruel…I felt suffering but I learned many things from it as well. Now when looking back, I feel it is a good exercise for my personal growth. (Participant 26, Interview 26)*

#### It is hard to go back

The goal of migration is socially constructed. Historically, Chinese people have perceived migration to the West as a desirable goal and going abroad is seen as “becoming gold-plated” and associated with increased social prestige [57]. Although few participants had been exposed to the *actual* experience of living abroad prior to emigrating, they anticipated that migration would result in a better life. However, a disconnection between what was expected and what actually occurred emerged after emigration [10].

*At the beginning, you feel immigration is a good thing, a road to happiness. But ultimately many people feel it is very difficult. (Participant 11, Interview 11)*

Indeed, the gains of emigration were also associated with difficulties, disappointments, costs, and losses. These negative aspects produced mixed feelings about migrating. Life in Australia may be better, but it is not necessarily better for every individual. Drawing comparisons between one life and another added to the ambivalence of the experience [10]. The dream of migration transformed into a dream of return after encountering the reality of living abroad.

*Most possibly I will stay here for a long period, and then go back to China to see whether there is a good chance for me. I really want to go back to China, but there are many factors influencing my decision. (Participant 25, Interview 25)*

There are several explanations of why participants would desire to return home. First, few immigrations are free of hardship. A tendency to over-emphasise memories of the positive aspects of the country of origin arise due to the challenges of the new country [52]. Although they were living in Australia, the nurses may have been emotionally attached to China [10]. Second, immigrants are in “a state of in betweenness” that makes them yearn for one place while living in another; i.e., they identify with home when abroad, and identify with abroad when at home [58]. Additionally, as the other, the participants were likely ambivalent about making Australia home. Thus, the participants aspired to return to “a place called home” where they felt comfortable and did not appear to be a foreigner. The dream of return may in fact be extremely important for immigrant identity and returning home is thus full of symbolic meanings [59].

Nonetheless, after experiencing the positive aspects of working as nurses in Australia, such as reasonable workloads, higher salaries, greater professional status, and increased autonomy, the participants were also reluctant to return to their old ways of life. It is not that the participants did not want to go back, but it was hard for them to do so [10].

*I’ve thought of going back to China but found it is very hard… If there is a good chance that I can develop my career and live a comfortable life, I will go back definitely. Because now I am away from home…Local people still treat me as Chinese and I cannot fit into the society here totally. (Participant 3, Interview 3)*

Emigration as a goal that is pursued at great cost, and, as a result, it is cherished when it is achieved. Because the participants had already endured the most difficult time in Australia and perceive that their situations were gradually improving, they did not want to forfeit their cherished accomplishment. It is also possible that the participants did not return despite dissatisfaction because once one has moved ahead, it is difficult to go backwards. Investments in homes and child education and the resulting community reflected a level of commitment that was difficult to abandon. Indeed, permanent return was more often a myth than a reality as the participants’ lives and jobs inevitably became embedded in the Australian context [10].

## Implications for Practice and Research

### Implications for practice

This study found that China-educated nurses considered immigration as a fulfilment of the goal of a better life. They were unprepared for the struggles that would accompany emigration. Unrealistic expectations predisposed the nurses to many hardships, disappointments, and frustrations. Therefore, providing Chinese nurses who wish to emigrate access to adequate and realistic information that ensures a balanced view of immigration life would be beneficial.

Another finding of this study was that the support provided to the China-educated nurses during their transition was inconsistent and inadequate. Moreover, although the participants considered dealing with unknowns to be their responsibilities, this does not mean that support was not needed or wanted. Indeed, the issue of support is much more complex than the most prevalent suggestions of the literature. More conversation and discussion are needed to promote mutual understanding and increase the efficacy of support services. One issue that remains to be addressed is how to create non-threatening work environments. Another concern is the negative connotation of support. A further issue is how to promote an inclusive culture that values rather than eliminates diversity. Given the circumstances of immigrant nurses (i.e., being situated as the other), merely offering some education in an effort to achieve this goal is inadequate.

### Implications for research

The findings of this study suggested that the husbands of the China-educated nurses experienced significantly diminished social status and employment prospects following immigration. We suggest that future studies that focus on the husbands and their experiences of settlement and the changing dynamics of families following immigration be conducted. Additionally, the concepts of reconciling and ambivalence should be further explored in immigration studies. Finally, a more in-depth analytical and theoretical focus is desirable in this research area.

## Conclusion

This study explored the experiences of China-educated nurses working in Australia. The core category developed in this study was the “reconciling different realities”. Realising refers to an awareness of the discrepancies between different realities. Struggling reflects the dilemmas of being the “middle position” and the experience of being “the other”. Reflecting is the process of making sense of the experience and rationalising the gains and losses associated with immigration. Through reflection, the participants arrived at a new level of realising and the process of reconciling continued. While these three phases were essential components of the experience, they did not always occur in sequence. In other words, the process of reconciling was non-linear. Over time, the participants attained some measure of resolution despite never reaching full reconciliation.

### Relevance to clinical practice

The findings of this study are not only informative to Chinese nurses who wish to immigrate but may also contribute to the implementation of more effective support services for immigrant nurses.
